# Physiotherapy Approach in Bilateral Parasymphysis Fracture With Bilateral Zygomaticomaxillary Complex Fracture: A Case Report

**DOI:** 10.7759/cureus.54897

**Published:** 2024-02-25

**Authors:** Neha M Chitlange, Tejaswini Fating

**Affiliations:** 1 Community Health Physiotherapy, Ravi Nair Physiotherapy College, Datta Meghe Institute of Higher Education and Research, Wardha, IND

**Keywords:** rocabado exercise, physiotherapy, trauma, zygomaticomaxillary complex fracture, parasymphysis fracture

## Abstract

The mandible occupies the lowest point on the face. When the lower face is struck by high blows with upward or obliquely directed force, the prominent bone of the face sustains severe damage. When combined with zygomaticomaxillary complex fractures, mandibular fractures, which frequently occur in parasymphysis, present a challenging clinical scenario. This combination often requires a collaborative strategy to ensure a proper diagnosis and all-encompassing care. In this case study, we discussed the alleged trauma case of a 30-year-old male who met with a road traffic accident due to a bike slip at around 8:30 p.m. on October 8, 2023, in Pulgaon. He was taken to a rural hospital and referred for further management. An orthopantomogram was done on investigation, and bilateral para-symphysis fracture and bilateral zygomaticomaxillary complex fracture were noticed. Later, the patient underwent surgery, where open reduction and internal fixation of the bilateral parasymphysis and right zygomaticomaxillary buttress fracture and closed reduction of the right zygomatic arch fracture under general anesthesia were made. Intermaxillary fixation was done. Then, he was referred to the physiotherapy department for therapeutic intervention. The rehabilitation goals were to maintain the strength of the afflicted muscles, regain full range of motion, minimize pain and edema, and gradually increase mobility. The patient was put on a four-week regimen. It was found that the patient reacted favorably to the treatment.

## Introduction

A common type of facial trauma is mandibular fractures, which account for 36-70% of all maxillofacial fractures [1]. These represent among the second-most prevalent facial injuries that trauma centers treat [2]. Mandibular fracture rates vary among nations and are subject to change over time [2]. Road traffic accidents mostly cause these, as they do in many other major cities and emerging countries. They pose a severe threat to India's public health [3]. A study in central India found that the male-to-female ratio of 3.7:1 corresponded to the highest incidence of mandibular fractures in men [3]. The most commonly fractured areas are the body, condyle, and angle. [4]. Fractures to the symphyseal/para-symphyseal area are less common, but the ramus and coronoid processes are barely impacted [4]. According to epidemiological studies, men between the ages of 21 and 30 are more likely to suffer mandibular fractures [5]. The most common reasons for mandibular fractures include car accidents, falls, and attacks [4]. The condylar region is the most frequently fractured area in car accidents; conversely, the symphysis is more commonly affected in motorcycle accidents [4]. The angle is likely the location that bursts most frequently in assault instances [4].

While zygomaticomaxillary complex fractures affect the upper jaw and zygoma, bilateral para-symphysis fractures damage the mandibular core area [6]. Zygomaticomaxillary complex fractures are clinically indicated by pain, swelling, bruising on the cheeks and eyelids, malar flattening, and palpable periorbital step-offs [7]. Furthermore, lockjaw, sunken eyes, double vision, and infraorbital nerve paresthesia are possible functional issues resulting from zygomaticomaxillary complex fractures [8]. Bilateral para-symphysis fractures may cause anomalies such as malocclusion and involuntary posterior tongue movements [8]. Treatment options for a mandible fracture can vary depending on the fracture's features and the surgeon's treatment selections [9]. These possibilities include non-operative therapy (such as a soft diet), closed reduction, and open reduction with internal fixation. Better treatment results were obtained for fractures with comminuted fragments when reconstruction plates were used [10]. One method for stabilizing and reducing mandibular fractures is called intermaxillary fixation [10].

The goal of physical treatment, particularly for parasymphysis with a zygomaticomaxillary complex fracture, is to restore range of motion and facial function in situations with facial trauma [11]. Physical therapy aims to help patients regain their capacity to move their lips, close their eyes, bite, and swallow [12]. As part of their after-surgery recovery, patients with facial trauma may have fractured bones, wounds to soft tissue, and significant soft tissue damage that need physical therapy [13]. The functional status and quality of life (QOL) of individuals with traumatic face fractures can be significantly enhanced by physiotherapy therapies [12]. Physiotherapy aims to maintain muscle strength, restore full ROM, minimize discomfort and swelling, and gradually boost mobility [13]. As a result, physiotherapy is crucial to the overall care of face trauma and aids in the recovery and well-being of those who are impacted.

## Case presentation

**Figure 1:**
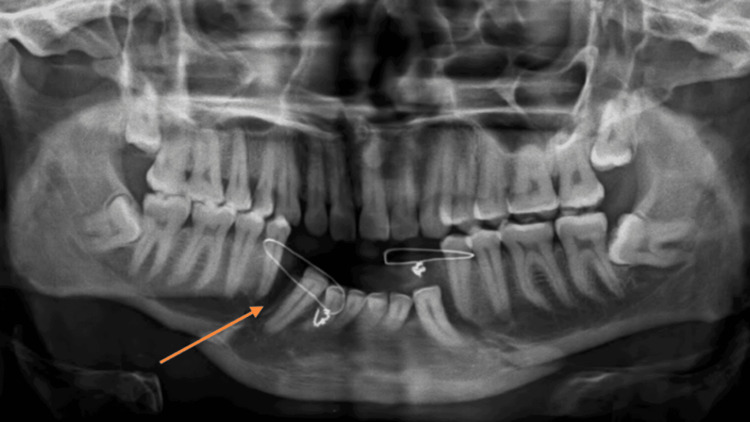
Pre-operative orthopantomogram The orange arrow indicates a bilateral para-symphysis fracture and a bilateral zygomaticomaxillary complex fracture.

**Figure 2:**
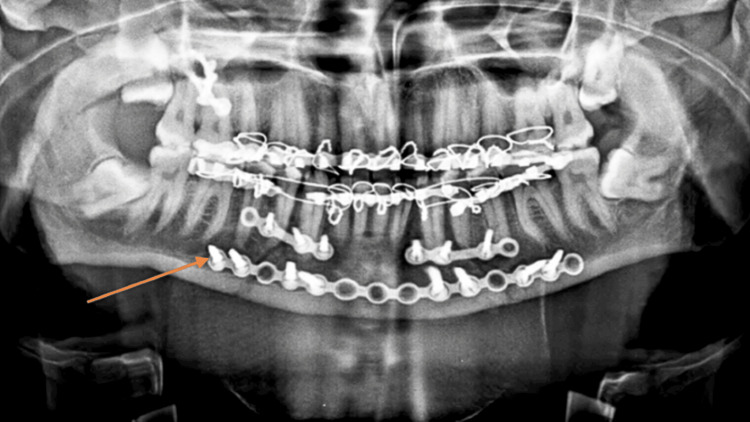
Post-operative orthopantomogram The orange arrow shows open reduction internal fixation of the bilateral parasymphysis and right zygomaticomaxillary buttress fracture and closed reduction of the right zygomatic arch fracture with intermaxillary fixation.

A 30-year-old male labors by occupation with a dominant right hand. He gave an alleged history of RTA due to a bike slip under the influence of alcohol at around 8:30 p.m. on October 8, 2023, in Pulgaon. After which, he had a loss of consciousness for 1-2 hours. He was taken to Rural Hospital Amravati by ambulance, where he was referred to a casualty for further treatment and management of contrast-enhanced computed tomography of the abdomen. He was diagnosed with blunt trauma with hemoperitoneum and a grade I right renal injury with adrenal hematoma. He was admitted to the surgery intensive care unit from 09/10/23 to 18/11/23, where medication like injection (Inj) Cfotaxime, Inj Piperacillin, Inj Metronidazole, Inj Pantoprazole, Inj Ondansetron, Inj Paracetamol, Tablet (Tab) Amoxicillin, and Tab Acetaminophen were administered. Then, from October 19 to October 23, he was admitted to the surgery ward for observation and was shifted to the cancer specialty hospital, Wardha, with a complaint of pain and swelling over both sides of the face. Pain was sudden in onset, had a dull-aching nature, and was aggravated by movement. He also gave a history of alcohol consumption dating back four years. On investigation, an orthopantomogram was done, which revealed a bilateral para-symphysis fracture and a bilateral zygomaticomaxillary complex fracture, as shown in Figure 1. On October 28, 2023, he underwent surgery, in which open reduction internal fixation of the bilateral parasymphysis and right zygomaticomaxillary buttress fracture and closed reduction of the right zygomatic arch fracture under general anesthesia were made, as shown in Figure 2. Intermaxillary fixation was done for two weeks. After surgery, the patient reported pain at the suture site, which was gradual in onset and dull aching in nature, cough, trismus, and restricted mouth opening. For this complaint, the patient was referred for physiotherapy. The timeline is shown in Table 1. ﻿

**Table 1:** Timeline RTA: Road Traffic Accident, AVBRH: Acharya Vinobha Bhave Rural Hospital; SICU: Surgery Intensive Care Unit; SGMCH: Siddhart Gupta Memorial Cancer Hospital

Date	Events
8/10/23	RTA
9/10/23	Visited AVBRH and was admitted to SICU
19/10/23	Shifted to the Surgery ward for observation
23/10/23	Admitted to SGMCH
24/10/23	Diagnosed and planned for surgery
28/10/23	Operated
29/10/23	Physiotherapy started

Clinical findings

The patient’s consent was obtained before the examination. The patient was assessed in a lying position with the heel elevated to 30 degrees. His neck was slightly flexed, his shoulder protracted, and his elbow and wrist were in extension bilaterally; his bilateral hip, knee, and ankle joints were in plantarflexion. There was no deformity observed. In the general examination, the patient was afebrile, had a heart rate of 86 beats/minute, a respiratory rate of 18 beats/minute, and a blood pressure of 128/80 mmHg. Ryle’s tube was present.

Head and neck examination

On examination, the face was grossly asymmetrical due to swelling over both sides of the face. Restricted jaw movements due to intermaxillary fixation. Tenderness in grade 2 was present over the bilateral para-symphysis region, extraorally over both sides of the malar area, and in the zygomatic arch. Jaw movements, like the opening and closing of the mouth, were altered. The mouth opening measured on the vernier caliper after intermaxillary fixation was removed (on the 15th day) is shown in Table 2. A cervical movement examination was done on week one and at the end of week two, as shown in Table 3. The cervical muscle isometric was weak. Cervical and shoulder muscle strength was reduced, as shown in Table 4. Postoperative medication is shown in Table 5. A detailed physiotherapy treatment is shown in Table 6. ﻿

**Table 2:** Mouth opening with a vernier calliper

Week 3	Week 4
1.5cm	5.5cm

**Table 3:** Range of motion

Variable	Movement	Week 1	Week 2
Cervical joint	Flexion	0-10^o^	0-35^o^
	Extension	0-10^o ^	0-30^o^
	Lateral flexion (Left)	0-5^o^	0-35^o^
	Lateral flexion (Right)	0-5^o^	0-35^o^
	Rotation (Left)	0-10^o^	0-45^o^
	Rotation (Right)	0-10^o^	0-45^o^

**Table 4:** Manual muscle testing (according to MMRC grading) MMRC: Modified Medical Research Council; 2: Full range of motion gravity eliminated; 3: Full range of motion against gravity; 4: Full range of motion against gravity with minimum resistance.

Joint	Muscle group	Week 1		Week 2	
		Left	Right	Left	Right
Cervical joint	Flexor	2	2	3	3
	Extensor	2	2	3	3
	Rotators	2	2	3	3
	Lateral flexor	2	2	3	3
Shoulder joint	Flexor	3	3	4	4
	Extensor	3	3	4	4
	Abductors	3	3	4	4
	Adductors	3	3	4	4
	Medial rotators	3	3	4	4
	Lateral rotators	3	3	4	4

**Table 5:** Post-operative medication Inj: Injection; gm: gram; IV: Intravenous; mg: milligram; TDS: Ter die sumendun (thrice daily); PCM: Paracetamol; NS: Normal saline; BD: bis in die (twice daily); HS: Hora somni (take at bedtime); Tab: Tablet

Medications	Dosage
Inj. Ceftriaxone S 1gm	IV Twice daily * 3 days
Inj. Metro 500mg	IV TDS*3 days
Inj. Pan 1 mg	IV Twice daily *3 days
Inj. PCM 1 gm in 100ml NS	IV Twice daily *3 days
Inj. Perinorm 10mg	IV BD*2 days
Inj. Chymoral forte	BD *7 days
Tab. Levocet 10 mg	HS * 7 days
Cavol steam inhalation	TDS
Protein powder with milk	150 gm /day

**Table 6:** Physiotherapy treatment reps: repetition

Phase	Physiotherapy goals	Physiotherapy treatment	Dosage
Week 1 – 2	To educate the patient	To guide the patient regarding the rehabilitation program and its effect. To make the patient aware of the preventive strategy during rehab.	Early ambulation, positioning and resumption of activities of daily life education
	To lessen oedema and pain.	Cryotherapy	10 minutes
	To avoid breathing difficulties.	diaphragmatic breathing	10 reps×1 sets of each exercise
		deep breathing	
		thoracic expansion exercise	
	To avoid slouched and forward-leaning postures.	door frame stretch	10 reps×1 sets of each exercise
		foam roller arching	
		shoulder blade squeezes	
	To enhance the mobility of the upper limbs.	Bilateral shoulder and cervical joint active range of motion	10 reps×1 sets of each exercise
Week 3–4	To improve oral opening and reduce trismus	mouth opening using ice cream sticks.	10 reps×1 sets of each exercise
		mouth blowing exercises	10 reps×1 sets of each exercise
		jaw deviation exercise	
		Mouth Puffing exercise	
	To treat trismus and strengthen the facial muscles	Oral motor exercises with resistance based	10 reps×1 sets of each exercise
		Goldfish exercise	6 times
		Thera-bite device for mouth-opening.	5 times
	To ease the tightness in the neck muscles.	Upper trapezius muscle stretching	5 times with 30 second hold
		Every session ended with cryotherapy.	10 minutes
	To increase upper limb muscular strength.	Muscle building exercises for the upper limb with 1litre bottle	10 reps×1 sets of each exercise

Suture examination

The pain was scored on the Numerical Pain Rating Scale, which was 8 and 6 by 10 on activity and rest. On inspection, a suture was seen in the symphysis region and was 10 cm long. There is no discharge or skin discoloration on the suture site. On palpation, the local temperature was raised. On postoperative day one, tenderness was three, i.e., the patient complained of pain and winces; on postoperative day 10, there was no tenderness.

Respiratory examination

**Figure 3:**
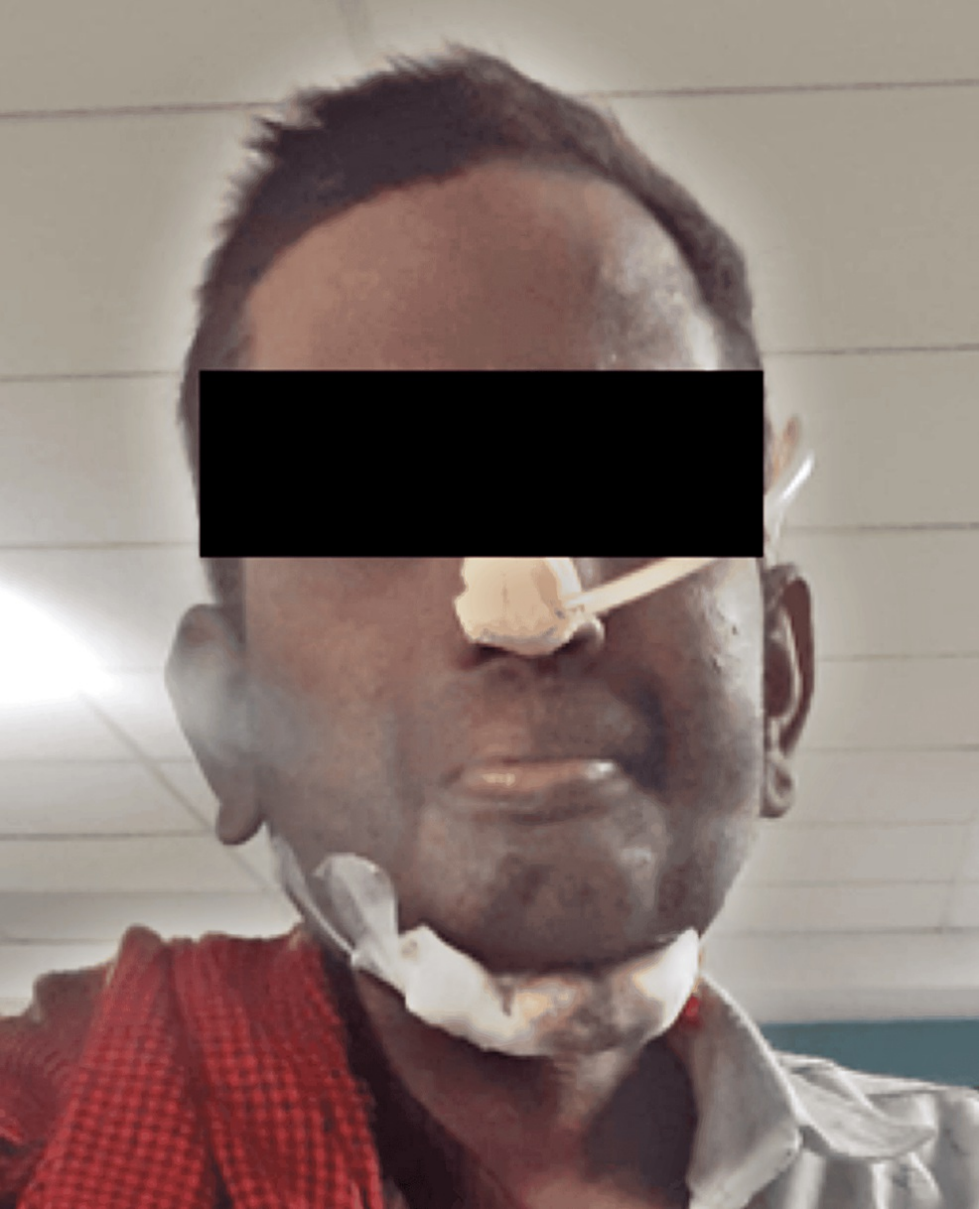
Deep breathing exercise

On inspection, no hollowness is seen, the anterior-posterior diameter is less than the transverse diameter, no chest deformity is seen, and the breathing pattern is abdomino-thoracic. On palpation, the trachea is placed centrally. Chest expansion is reduced at the axillary and xiphoid levels. On auscultation, crepitus is present in the lower zones, and air entry is decreased in the upper zone. The patient performing deep breathing exercises is seen in Figure 3.

Home exercise program

**Figure 4:**
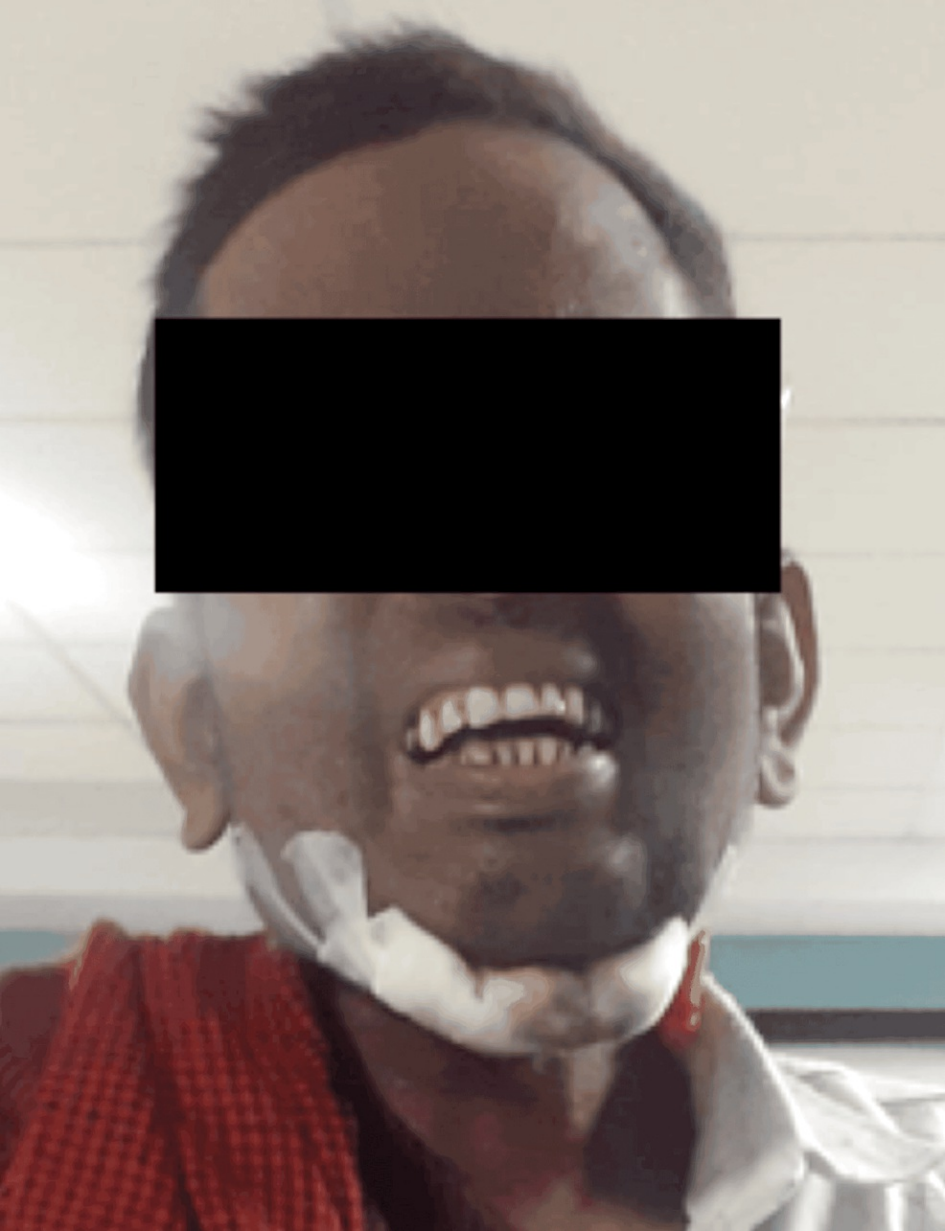
Patient performing the Rocabado exercise

After being discharged, the patient was taught how to complete the exercises in the home program, continuing each practice with the appropriate number of repetitions. Goldfish and Rocabado training (six exercises, six times) were taught to the patient, as seen in Figure 4. The pain was lessened, and there was an increase in mouth opening.

## Discussion

With the help of this study, the body of evidence supporting physiotherapy interventions for treating maxillofacial disorders can be increased. The quality, dosages, and types of exercise used in earlier research examining the effects of post-mandibular fracture exercise regimens varied. Additionally, all of them were performed by surgeons without any interventions or demonstrations of physical therapy [14]. Treatment for trismus requires a well-planned physical therapy intervention. A case study by Rasotra shows how physical therapy significantly improved a patient's trismus [15].

Physical therapy, which tries to lessen jaw and neck pain while increasing the range of motion and promoting exercise to maintain healthy function, is one of the most widely used treatments for temporomandibular disorder, according to a study by Armijo-Olivo et al. [16]. Wang et al. found that following an average of four weeks of physical therapy intervention, patients showed statistically significant improvements in cervical range of motion, pain relief, physical performance measures, and the degree of disability [17]. Physical therapy helped patients who had suffered facial trauma and had acute neck sprains [18]. Active mobilization exercises promote early healing of fracture sites and allow for optimal mouth-opening functional ranges [19]. When treating patients with limited mouth openings, physiotherapy is essential. Goldfish and Rocabado exercises are two examples of interventions used in this regard. As a result, the patient's condition improves, and they become more self-sufficient in performing daily tasks [20].

Nevertheless, there is a shortage of research on using physiotherapy to treat mandibular fractures [12]. Individuals with mandibular fractures benefit from increased functional abilities thanks to physical therapy. In this study, we spoke about a 30-year-old male patient who allegedly suffered injuries after falling from his bike and visiting the hospital. He was found to have bilateral zygomaticomaxillary complex fractures as well as bilateral para-symphysis fractures during an examination. Operationally, it was treated with open-reduction internal fixation using screws and titanium plates. A physical therapy regimen is initiated on his second postoperative day. It continues until the patient resumes his pre-surgical activities, including eating, talking, chewing, and cervical motions. The four-week intervention markedly enhanced the patient's quality of life.

## Conclusions

Our therapeutic approach successfully treated the patient's trismus, restricted mouth opening, pain, and facial swelling, significantly improving the patient's overall quality of life. One of the most crucial strategies to improve patient outcomes and lower the risk of postoperative complications was incorporating rehabilitation into the treatment plan. Though research on rehabilitation for mandibular dysfunction is limited, what is known from the studies is intriguing, with one notable discovery being the creation of a fantastic four-week regimen. A patient with challenging zygomaticomaxillary fractures and bilateral parasymphysis responded well to our treatment approach. This significant new study recently highlighted the benefits of physical therapy for managing mandibular injuries. This case report highlights the necessity of an all-encompassing approach to optimize patient recovery and well-being in challenging circumstances.
